# A production planning model considering uncertain demand using two-stage stochastic programming in a fresh vegetable supply chain context

**DOI:** 10.1186/s40064-016-2556-z

**Published:** 2016-06-22

**Authors:** Jordi Mateo, Lluis M. Pla, Francesc Solsona, Adela Pagès

**Affiliations:** Computer Science Department and INSPIRES, University of Lleida, Jaume II 69, 25001 Lleida, Spain; Department of Mathematics, University of Lleida, Jaume II 73, 25001 Lleida, Spain

**Keywords:** Production planning, Supplier selection, Fresh vegetable supply chain, Two stage mixed 0–1 models, Lagrangian relaxation, Parallel computing

## Abstract

Production planning models are achieving more interest for being used in the primary sector of the economy. The proposed model relies on the formulation of a location model representing a set of farms susceptible of being selected by a grocery shop brand to supply local fresh products under seasonal contracts. The main aim is to minimize overall procurement costs and meet future demand. This kind of problem is rather common in fresh vegetable supply chains where producers are located in proximity either to processing plants or retailers. The proposed two-stage stochastic model determines which suppliers should be selected for production contracts to ensure high quality products and minimal time from farm-to-table. Moreover, Lagrangian relaxation and parallel computing algorithms are proposed to solve these instances efficiently in a reasonable computational time. The results obtained show computational gains from our algorithmic proposals in front of the usage of plain CPLEX solver. Furthermore, the results ensure the competitive advantages of using the proposed model by purchase managers in the fresh vegetables industry.

## Background

Nowadays, little by little are appearing studies in which operations research are applied to solve agricultural problems for the agroindustry. See Plà et al. (2013) to review some opportunities and prospectives in this field. Some examples can be found for different sectors in the current literature, for example, a review of models for transport planning for the fresh fruit supply chain is presented in Soto-Silva et al. (2015). A collection of a variety of models applied to the agroindustry is included in Plà-Aragonés (2015), while Ahumada and Villalobos (2009a) review several agrifood supply chain models.

The fresh vegetable industry is a very important economic activity in Spain. Novel trends in Spain are related to the consumption of quality-local products. Many grocery shops prefer to add value to their products offering local fresh vegetables (fresh vegetables produced in the surroundings) rather than importing the same vegetables from other countries or regions at a lower cost. Green and sustainable products of proximity are labels associated many times to this kind of products well appreciated by a more awareness consumer. Nowadays, the quality of a vegetable is not only measured through its appearance or flavour, but also by where it has been grown or how much traceable information can be offered to the final customer.

In this context, professional purchase managers in these brands have to deal with the problem of choosing a set of farms to contract production from season to season so as to minimize the overall cost while satisfying the future demand. In this way, these grocery shops are able to sell tasty and healthy locally-produced vegetables with full traceability, making the activity sustainable over time with beneficial effects for the local economy.

However, selecting a farm to grow vegetables on is a mid/long-term decision. The decision has to be made beforehand with the uncertainty of the future behaviour or conditions of production and demand for the following season. Thus, a two-stage model with a “here and now” strategy is needed to deal with this kind of problem. For more information about stochastic programming see Birge and Louveaux (2011), Shapiro et al. (2014) and Prékopa (2013).

The current literature on fresh vegetable supply chain contains several examples of successful implementations which are focused on the maximisation of the total revenue for the grower. For instance, see Ahumada and Villalobos (2009b). However, there are no successful model focused on minimising the costs in a competitive market where companies can rent or contract farms to grow up fresh vegetables. The model proposed in this paper is oriented to grocery shops, big retailers and distributors inside this particular context.

The objectives of the present study are:

- Design a model capable of dealing with the decision of choosing the best set of farms to contract their production in the fresh vegetable production context.
- A parallel algorithm is proposed to solve the model in order to alleviate computational load in serial procedures, to reduce computational time, and also to make possible the usage of the model by purchase managers in the agroindustry.

One of the main contributions of this paper is the proposal of a production planning model by a two-stage model capable of dealing with the problem of selecting suppliers in a fresh vegetable supply chain. This production planning model is based on the adaptation of the linear Uncapacited Facility Location Problem (Cornuejols et al. 1983; Erlenkotter 1978), in which facilities are replaced by production contracts with suppliers. The model presented is very flexible and can be easily adapted to other financial contexts with similar characteristics and restrictions.

Numerous methods have been proposed to solve the Uncapacited Facility Location Problem. For example, neighbourhood search heuristic (Ghosh 2003), Lagrangian based heuristic (Beasley 1993), specific heuristics (Avella et al. 2009), benders decomposition (de Camargo et al. 2008), branch-and-price (Ro and Tcha 1984), tabu search (Al-Sultan and Al-Fawzan 1999), Lagrangian relaxation (Wu et al. 2015), etc.

Another important contribution of this paper is the proposal of an algorithmic approach to solve huge real instances of two-stage mixed 0–1 models using parallel computing paradigms. This work presents the parallelization of the Subgradient Method proposed in Escudero and Garín (1984). The combination of Lagrangian relaxation and parallel computing techniques makes this model flexible for purchase managers in their daily tasks. Current research seems to validate the potential of parallel computing in stochastic programing. Thus, this paper presents a novel way of dealing with production planning by farm selection inside a supply chain in the vegetable production environment.

## A two stage model for uncertain fresh vegetable production planning

This model is adapted from the classic uncapacited facility location problem (Cornuejols et al. 1983; Erlenkotter 1978). From here on, *S2FVPP* is used as the model name.

The objective of the proposed model is to evaluate the available fresh vegetable farms and determine the ones that minimize the overall cost so as to satisfy the uncertain demand of the potential customers. The model is intended for cooperatives or private companies to accept or recommend farms to be part of them or to distributors to agree production contracts.

**Fig. 1:**
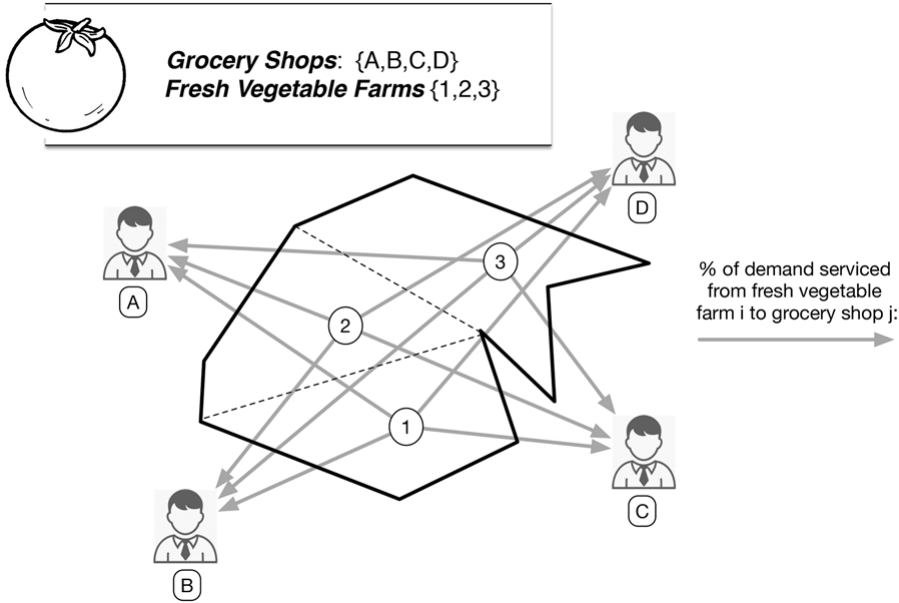
The uncapacited facility location problem applied to the selection of the most suitable fresh vegetable farms as provider to the fresh vegetable supply chain servicing to specific providers

The parameters and the decision variables used to formulate the model are listed in Table 1. The *S2FVPP* configuration decisions consist of choosing whether to use a fresh vegetable farm to grow fresh vegetables or not. A binary variable is associated with the selection of these fresh vegetables farms in such a way that $y_{i}=1$ whether the fresh vegetable farm *i* is used to grow up the fresh vegetables; otherwise $y_{i}=0$. Let $x_{ij}$ denote the fraction of demand serviced from farm *i* to the customer *j* under a specific stochastic scenario $\omega$. Furthermore, the cost of growing a unit of fresh vegetable in the fresh vegetable farm *i* is represented by $cgv_{i}$, and the last but not least, the cost of serving a customer *j* from fresh vegetable farm *i* under the scenario $\omega$ is denoted by $csv_{ij}^{\omega }$. Furthermore, a customer cannot be served from a fresh vegetable farm unless we contract its production, see (1e). Besides, each customer *j* must be full served so (1b) is needed in the model. Moreover, the model must ensure that the total amount of demand is satisfied by the final farm selection, see (1c). Last but not least, the amount of demand served for each fresh vegetable farm can not exceed its maximum productivity, see (1d). Finally, (1f) defines the variable $y_i$ as binary (Fig. 1) shows this model.

1a $$\begin{aligned} (S2FVPP) \quad&min\,\,\sum _{\omega \in {\varOmega }} \pi ^{\omega } \bigg [ \sum _{i \in F} \sum _{j \in C} x_{ij}^{\omega } csv_{ij}^{\omega }\bigg ] + \sum _{i \in F} cgv_{i} y_{i} \end{aligned}$$

1b $$\begin{aligned} s.t.: \quad&\sum _{i \in F} x_{ij}^{\omega } = 1, \quad \forall j \in C,\quad \forall \omega \in {\varOmega } \end{aligned}$$

1c $$\begin{aligned}&\sum _{i \in F} y_{i}s_{i}r^{\omega }_{i} \ge \sum _{j \in C} d^{\omega }_{j}, \quad \forall \omega \in {\varOmega } \end{aligned}$$

1d $$\begin{aligned}&\sum _{j \in C} d_{j}^{\omega }x_{ij}^{\omega } \le r^{\omega }_{i}s_{i}, \quad \forall \omega \in {\varOmega },\quad \forall i \in F \end{aligned}$$

1e $$\begin{aligned}&0 \le x_{ij}^{\omega } \le y_{i},\quad \forall i \in F,\quad \forall j \in C,\quad \forall \omega \in {\varOmega } \end{aligned}$$

1f $$\begin{aligned}&y_{i} \in [0,1], \quad \forall i \in F \end{aligned}$$

**Table 1:** Notations used in the mathematical parameters

*F*	Set of farms available in our production field
${\varOmega }$	Set of different uncertain scenarios
*C*	Set of different potential customers for our vegetables
$y_{i}$	Represents whether farm *i* is used or not
$x_{ij}^{\omega }$	% of demand serviced from farm *i* to customer *j* under scenario $\omega$
$csv_{ij}^{\omega }$	Cost of servicing the customer *i* from farm *j* under scenario $\omega$
$cgv_{i}$	Cost for growing a vegetable on farm *i*
$d_{j}^{\omega }$	Demand of customer *j* under scenario $\omega$
$r_{i}^{\omega }$	Profitableness per hectare of farm *i* under scenario $\omega$
$s_{i}$	Surface in hectares of farm *i*
$\pi ^{\omega }$	Represents the probability of scenario $\omega$

Problem (1), called *S2FVPP*, is expressed in compact representation, which is equivalent to its equivalent deterministic model (DEM), that, in the splitting variable representation, can be expressed as:

2a $$\begin{aligned} (S2FVPP) \quad&min \quad \sum _{\omega \in {\varOmega }} \pi ^{\omega } \bigg [ \sum _{i \in F} \sum _{j \in C} x_{ij}^{\omega } csv_{ij}^{\omega } + \sum _{i \in F} cgv_{i} y_{i}^{\omega } \bigg ] \end{aligned}$$

2b $$\begin{aligned} s.t.: \quad&\sum _{i \in F} x_{ij}^{\omega } = 1, \quad \forall j \in C, \quad \forall \omega \in {\varOmega } \end{aligned}$$

2c $$\begin{aligned}&\sum _{i \in F} y^{\omega }_{i}s_{i}r^{\omega }_{i} \ge \sum _{j \in C} d^{\omega }_{j}, \quad \forall \omega \in {\varOmega } \end{aligned}$$

2d $$\begin{aligned}&\sum _{j \in C} d_{j}^{\omega }x_{ij}^{\omega } \le r^{\omega }_{i}s_{i}, \quad \forall \omega \in {\varOmega },\quad \forall i \in F \end{aligned}$$

2e $$\begin{aligned}&y_{i}^{\omega } - y_{i}^{\omega +1} \le 0, \quad \forall \omega \in 1 \ldots {\varOmega } -1,\quad \forall i \in F \end{aligned}$$

2f $$\begin{aligned}&y_{i}^{{\varOmega }} - y_{i}^{1} \le 0, \quad \forall i \in F \end{aligned}$$

2g $$\begin{aligned}&0 \le x_{ij}^{\omega } \le y_{i}^{\omega }, \quad \forall i \in F,\quad \forall j \in C,\quad \forall \omega \in {\varOmega } \end{aligned}$$

2h $$\begin{aligned}&y_{i} \in [0,1], \quad \forall i \in F \end{aligned}$$

Note that in the splitting variable model Problem (2), the first stage decisions are replicated for each scenario. The non-anticipativity constraints (NAC), see (2e) and (2f), are needed to make the problem equivalent to Problem (1). NAC constraints are expressed in that form to avoid the use of non-negative vectors of Lagrangian multipliers in the dualization of equality constrains. The advantages of dealing with the splitting variable model is the possibility of decomposing it into different independent scenarios and solving them using the Lagrangian decomposition (Escudero and Garín 1984).

## Lagrangian relaxation

The Lagrangian relaxation (LR) of the *S2FVPP* for a given non-negative vector of weight $\mu _{y} = (\mu _{y_{1}},\ldots , \mu _{y_{|F|}})$ refers to the mixed 0–1 *LR* minimization Problem presented in this section.

3a $$\begin{aligned} (LR)\quad&min \quad \sum _{\omega \in {\varOmega }} \pi ^{\omega } \bigg [ \sum _{i \in F} \sum _{j \in C} x_{ij}^{\omega } csv_{ij}^{\omega } + \sum _{i \in F} (cgv_{i} + (\mu _{i}^{\omega } - \mu _{i}^{\omega -1})) y_{i}^{\omega } \bigg ] \end{aligned}$$

3b $$\begin{aligned} s.t.:\quad&\sum _{i \in F} x_{ij}^{\omega } = 1, \quad \forall j \in C,\quad \forall \omega \in {\varOmega } \end{aligned}$$

3c $$\begin{aligned}&\sum _{i \in F} y^{\omega }_{i}s_{i}r^{\omega }_{i} \ge \sum _{j \in C} d^{\omega }_{j}, \quad \forall \omega \in {\varOmega } \end{aligned}$$

3d $$\begin{aligned}&\sum _{j \in C} d_{j}^{\omega }x_{ij}^{\omega } \le r^{\omega }_{i}s_{i}, \quad \forall \omega \in {\varOmega }, \quad \forall i \in F \end{aligned}$$

3e $$\begin{aligned}&0 \le x_{ij}^{\omega } \le y_{i}^{\omega }, \quad \forall i \in F, \forall j \in C, \quad \forall \omega \in {\varOmega } \end{aligned}$$

3f $$\begin{aligned}&y_{i} \in [0,1], \quad \forall i \in F \end{aligned}$$

It can be shown that Problem *LR* is a relaxation of Problem (2) because:

- The feasible set of Problem *LR* contains the feasible set of Problem *S2FVPP*.
- For any feasible solution (*x*, *y*) from Problem *S2FVPP*, and also any positive $\mu$, the solution of Problem *LR* is a lower bound on the optimal value of Problem *S2FVPP*, thus $Z_{LR} \le Z_{S2FVPP}$.

The Problem $LR(\omega )$ provides a suitable structure to be decomposed in scenarios ($\omega$).

4a $$\begin{aligned} (LR(\omega )) \quad&min \quad \pi ^{\omega } \bigg [ \sum _{i \in F} \sum _{j \in C} x_{ij}^{\omega } csv_{ij}^{\omega } + \sum _{i \in F} (cgv_{i} + (\mu _{i}^{\omega } - \mu _{i}^{\omega -1})) y_{i}^{\omega } \bigg ] \end{aligned}$$

4b $$\begin{aligned} s.t.:\quad&\sum _{i \in F} x_{ij}^{\omega } = 1, \quad \forall j \in C \end{aligned}$$

4c $$\begin{aligned}&\sum _{i \in F} y^{\omega }_{i}s_{i}r^{\omega }_{i} \ge \sum _{j \in C} d^{\omega }_{j} \end{aligned}$$

4d $$\begin{aligned}&\sum _{j \in C} d_{j}^{\omega }x_{ij}^{\omega } \le r^{\omega }_{i}s_{i}, \quad \forall i \in F \end{aligned}$$

4e $$\begin{aligned}&0 \le x_{ij}^{\omega } \le y_{i}^{\omega }, \quad \forall i \in F, \quad \forall j \in C \end{aligned}$$

4f $$\begin{aligned}&y_{i} \in [0,1], \quad \forall i \in F \end{aligned}$$

One of the most common approaches to solving the Lagrangian relaxation is the subgradient method (Fisher 2004; Escudero et al. 2004; Escudero and Garín 1984), known as a general-purpose method. It is used often to solve generic non-smooth convex optimisation problems. Hereafter, the question under discussion is whether or not a parallel implementation of Lagrangian decomposition using the subgradient method is suitable to solve efficiently models similar to the one proposed in this work.

## Parallel Lagrangian decomposition

In this section, a parallel implementation of the Lagrangian decomposition method is proposed so as to gain computational efficiency in the resolution of problems such as model *S2FVPP*; see Problem (1). A serial implementation of Lagrangian decomposition using the subgradient method for dealing with two-stage stochastic mixed 0–1 models was presented and proposed in Escudero and Garín (1984).

The underlying argument in favour of designing a parallel implementation of Lagrangian decomposition using the subgradient method (**pSM**), is the independence between the problems generated by the scenario decomposition of model *LR*; see Problem (3). Furthermore, the operations performed by the subgradient method in each iteration are suitable to be executed in a parallel context too. Thus, the effort made in this work was focused on designing a parallel version of the subgradient method presented in Escudero and Garín (1984).

The parallel version **pSM** is identical to the serial version, with additional coding for shared memory data and synchronisation steps among the available computing cores. Instead of running a single computation task, the parallel implementation is able to run as many tasks as cores in the computing node are available.

The parallel algorithm proceeds to update first the objective function of the model *LR*($\omega$) from scenario 1 to scenario ${\varOmega }$ with the value $\mu ^{k}_{\omega }$, where *k* represents the current iteration of the method and $\omega$ represents a specific scenario, $\omega \in {\varOmega }$. Then, these updated *LR*($\omega$) problems are solved concurrently. Each thread, stores information about the solution such as the values of the variables $x_{ij}^{\omega k}$ and $y_{i}^{\omega k}$ inside the matrix structures, which belong to shared memory. The execution of this step does not represent any synchronisation problem in shared-memory environments. Since one of the dimensions of these matrices represents the assigned scenario, the threads, even executed in different cores, will not overwrite the same solution. After this step, **pSM** reduces all the partial solution of *LR*($\omega$), $z_{LR(\omega )}^{k}$, in the summatory $z_{LR}^{k}$. Hence, in this point, it is premised on the presumption of Eq. 5.

5 $$\begin{aligned} z_{LR}^{k} = \sum _{\omega =1}^{{\varOmega }}{z_{LR(\omega )}^{k}}. \end{aligned}$$

In a reduce operation, a private copy for each variable is created for each core. At the end, the reduction operation (sum) is applied to all private copies of the shared variable, and the final result is written in the global shared variable. Next, **pSM** computes all the subgradients, $S^{k}$, taking into account the solution of the first stage variables $y_{i}^{\omega k}$, the computation is performed using Algorithm 1.

The pseudo code of Algorithms 1 and 2 uses the notation of OpenMP to represent the shared-memory parallel approach. 
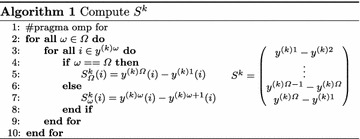


There is overwhelming evidence that two threads are accessing concurrently to the same memory region, because not only the values of $y^{(k)\omega }$ in the first stage belonging to a specific thread are needed to compute the Subgradient, but also the values of the solution of the following thread, $y^{(k)\omega +1}$. The basic premises of parallel shared memory paradigm is that the same memory region can be read concurrently for multiple threads if and only if no one writes on this memory region. Thus, the algorithm does not end in any memory exception.

Once all the subgradients are computed, **pSM** needs to evaluate in a single thread the status of the algorithm at iteration *k* using the current global solution. This process consists on checking whether the algorithm is able to improve the solution in future iterations. The criteria used for taking this decision are deeply explained in Escudero and Garín (1984). After this phase, in case that the algorithm improve the current solution, some convergence parameters are updated so as to boost the ending of the method. For detailed information about the choice and the improvement of these parameters, check Escudero and Garín (1984) too.

Following these serial steps, **pSM** updates the matrix $\mu ^{k+1}$ this procedure is described in Algorithm (2). 
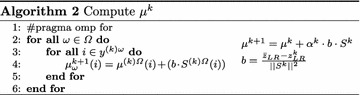


There is no need to argue about the correctness of Algorithm (2). Note that *b* is fixed for all threads. Thus, this value is computed by a single thread and stored in the shared memory in order to be accessible for all threads. Moreover, this value is computed in the previous step, just before checking the stopping criteria. ${{\bar{z}}_{LR}}$ represents an upper bound of the solution value of *S2FVPP*, see Problem (1).

Once the matrix $\mu ^{k+1}$ is updated for all threads, **pSM** goes to the next iteration, $k=k+1$.

The current literature on Lagrangian decomposition abounds with different examples of small modifications to improve the behaviour and the convergence of the method. The implementation of **pSM** takes advantage of the introduction of the scenario cluster concept.The proposed method is able to deal with scenario clusters, see Escudero et al. (2004).

**Fig. 2:**
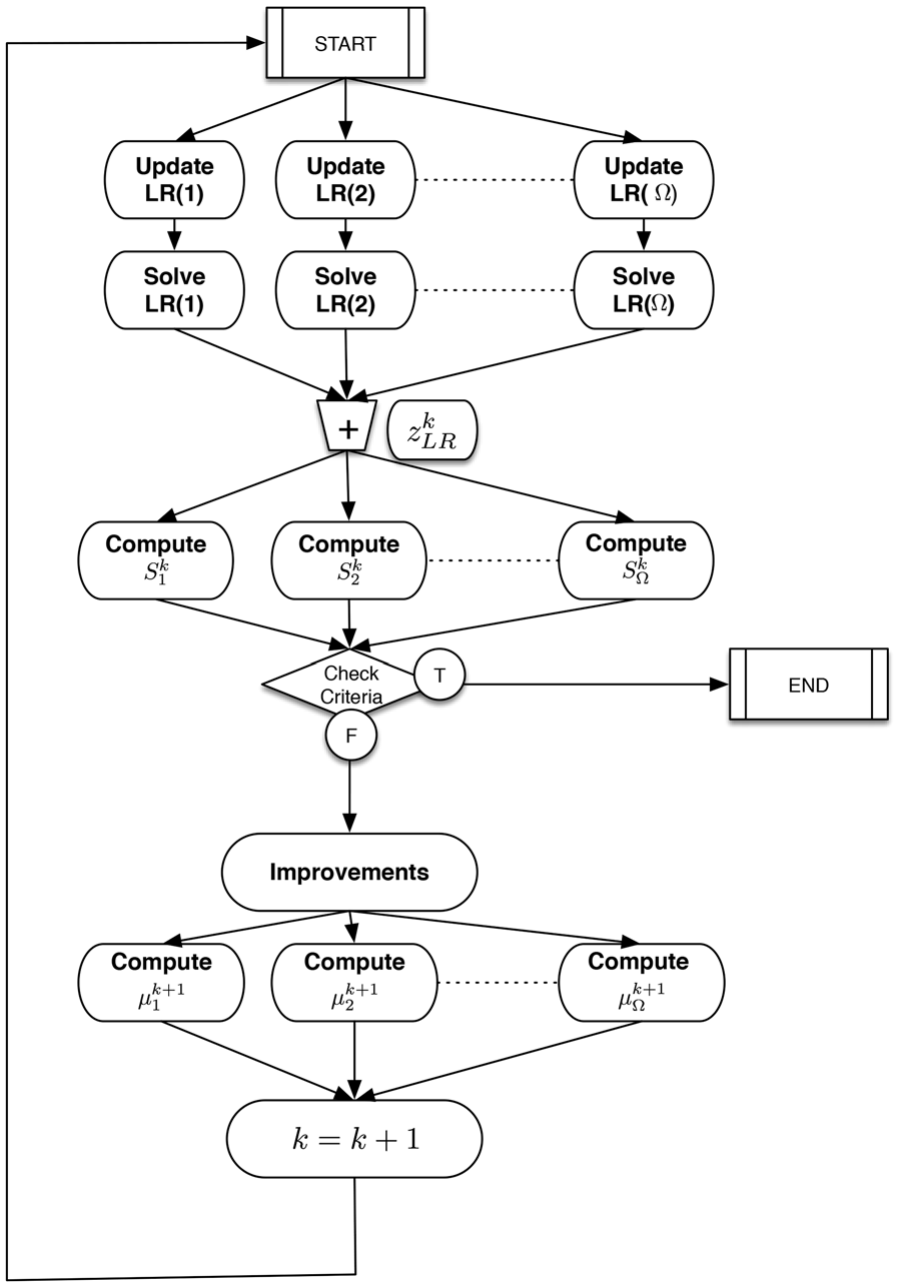
Parallel scheme for the Lagrangian decomposition method using the subgradient method in a shared memory context

Figure 2 shows the proposed **pSM** scheme. This scheme summarises the iterative process and highlights the steps realised in parallel by all available threads and the ones realised in serial by a single thread.

## Case study

In this section, a real study for *S2FVPP* instance is studied. A local chain of grocery shops is dealing with the problem of supplying tomatoes, grown by locally producers, at the minimum cost for the next year. Thus, the aim is to determine which tomato farms have to be contracted this season to satisfy future demand with local products.

**Fig. 3:**
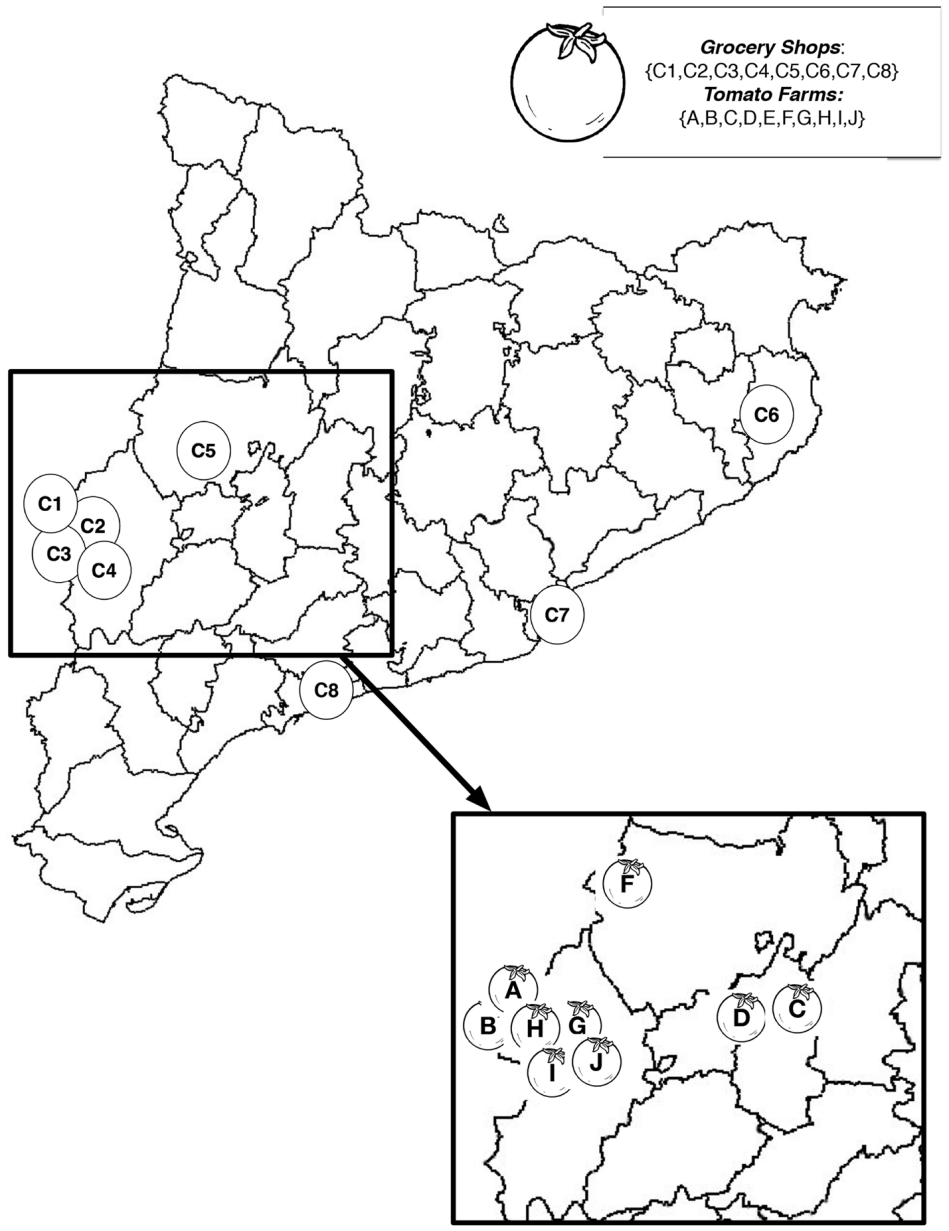
This graph represents the local chain distribution and tomato farms location in Catalonia region, for the case study

Moreover, the local chain of grocery shops is made up of eight shops {C1–C8} and has to select tomato farms {A–J} to fulfil the future demand. These shops and tomato farms are distributed throughout Catalonia, see Fig. 3. This map represent the location of each shop and farm, approximately.

The main characteristics of the tomato farms used in this study are summarised in Table 2. *Farm* represents the name of the farm, *Location* indicates the place and *Hectares* the land surface. Quality field ranges from 1 to 10, where 1 represent the lowest quality. This index is computed using both the knowledge of historical data for past seasons and customers feedback information. Note that in Table 2, farms {A, D} has not quality index (−), representing that neither historical data nor customers feedback information are recorded for these farms. Besides, each *cgv* coefficient is computed taking into account the quality index and the size of the yields.

**Table 2:** Summary stored in the data center with the main information for available tomato farms

Farm	Location	Hectares	Quality	*cgv* (0.09 €/kg)
A	Torreserona	0.005	–	9
B	Torreserona	0.008	8	8
C	Tàrrega	0.008	8	8
D	Anglesola	0.012	–	9
E	Mollerusa	0.016	7	18
F	Alfaràs	0.012	9	12
G	Torrefarrera	0.018	7	18
H	Alpicat	0.025	7	25
I	Alpicat	0.003	8	5
J	Torrefarrera	0.025	6	30

Scenarios are built considering production, demand and cost uncertainty of servicing each shop. By this way, 3 different scenarios are generated: poor, fair and boom with probability 0.22, 0.70 and 0.08 respectively. Table 3 shows the variation of the demand at each shop under the 3 scenarios considered in this study. Besides, the yield per hectare is assumed to be fixed. The scenario values considered are 50,000, 72,000 and 80,000 kg/ha for poor, fair and boom respectively.

**Table 3:** Information about the demand under each specific scenario

Name	Demand (kg)		
	Poor	Fair	Boom
C1	350	400	450
C2	200	300	350
C3	275	300	325
C4	250	325	400
C5	75	150	200
C6	150	250	400
C7	400	650	800
C8	250	300	350

The main features of the grocery shops used in this study are summarised in Table 4. *Grocery Shop* represents the name of the grocery shop and *Location* indicates the place. The *Serving Cost*; *csv*, is computed using information related with the transport and delivery costs estimated for each pair of tomato farm and grocery shop. Table 4 describes the cost of service under the fair scenario.

**Table 4:** Main information about the grocery shops

Grocery shop	Location	Serving cost per unit (0.09 €/kg)									
		A	B	C	D	E	F	G	H	I	J
C1	Lleida	5	5	10	10	7	10	6	6	6	6
C2	Lleida	5	5	10	10	7	10	6	6	6	6
C3	Lleida	6	6	11	11	8	11	7	7	7	7
C4	Lleida	5	5	10	10	6	10	6	6	6	6
C5	Balaguer	7	7	6	6	8	4	7	7	7	7
C6	Girona	30	30	20	22	25	37	28	28	28	28
C7	Barcelona	25	25	15	18	20	30	24	24	24	24
C8	Tarragona	16	16	10	11	20	25	16	16	16	16

Highlighting the serving cost *csv* under fair scenario per unit (0.09 €/kg) of demand

**Fig. 4:**
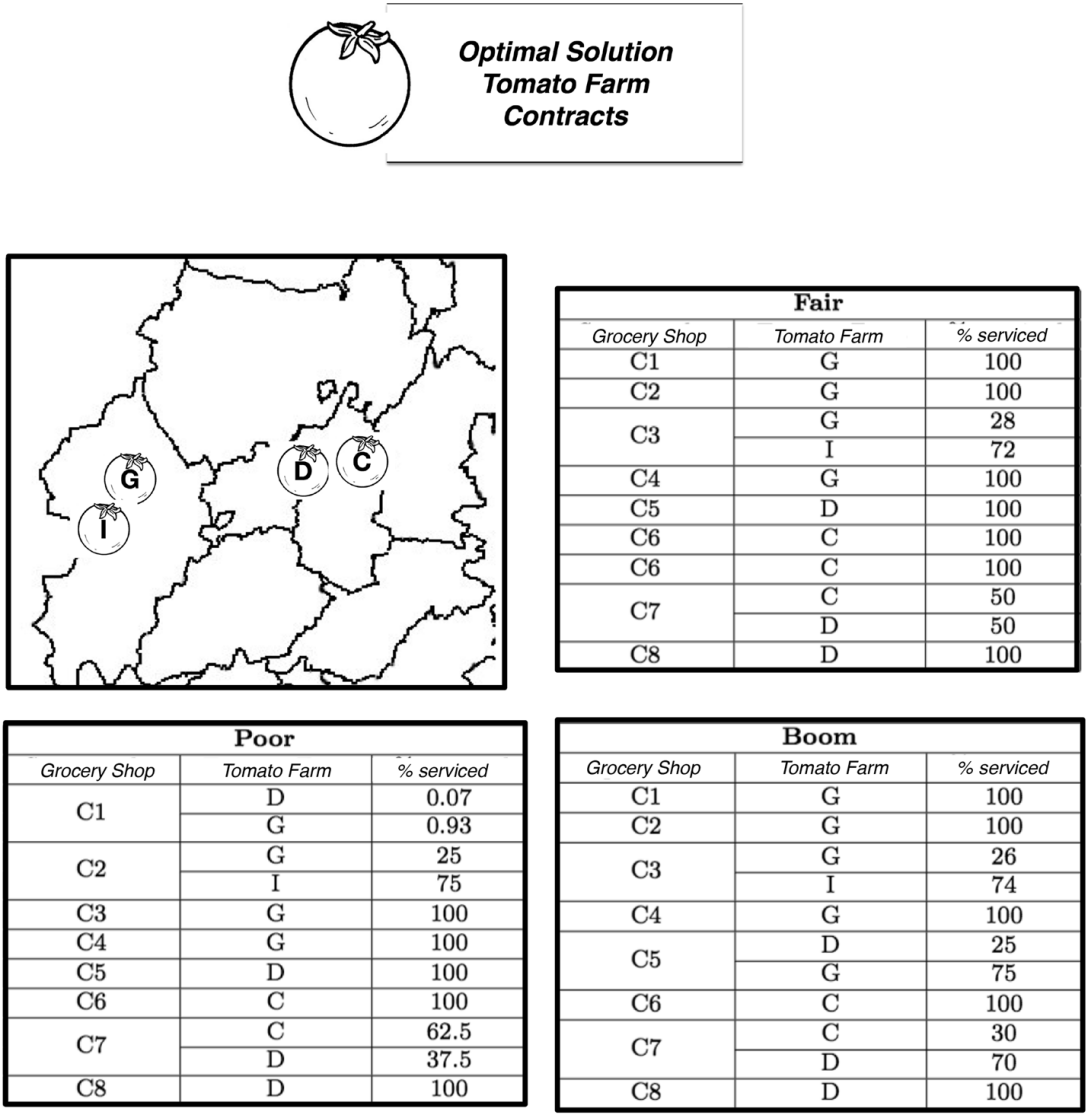
Tactical decisions for farm election. Percentages serviced under each scenario

Figure 4 shows the results and tactical decisions of the proposed model. These results show the farms selected to be used the following season. The selection of these farms minimizes the overall cost into 132.973. The proposed model is shown to deal with that kind of problem efficiently.

### Practical implications

The S2FVPP model presented in this paper is aimed at practical application for the fresh vegetable agri-food industry. There is overwhelming evidence corroborating the idea that industry needs to understand the model before rely on it. Choosing the best farms to contract production is a critical decision in order to design an efficient model to select suppliers and explore alternative solutions by purchase managers. This position allow the manager to gain knowledge about the range of prices he can fix on contracts considering the uncertainty represented by each scenario. Moreover, decisions makers are able to use the huge amount of big data gathered from their industrial context to feed the model with accurate coefficients for *csv* and *cgv* and enlarging the number of scenarios to be considered. Historical data sets for past seasons, consumers feedback, traceability, transport cost, among others, can be used to model these input parameters. Furthermore, the stochasticity of the model introduces the market uncertainty and makes possible to improve predictions about the future trends so as to make better decisions in the whole agri-food business context.

Given the interest of selling local fresh vegetables, the model helps purchase manager to choose the best fresh vegetable farms to sign production contracts and hence fulfil final consumers demand. By the analysis of the optimal solution, the industry can negotiate production contracts with the more suitable farms. In contrast, purchase managers without the model have to trust only on their expertise in the field in order to decide whether or not selecting a farm as supplier.

The model presented is a powerful tool capable of dealing with huge amount of information and a number of scenarios coping with the uncertainty of future fresh vegetable production to make the best decision. Much of the current solutions deals with the minimization of the global cost of choosing the most suitable neighbouring farms to supply products. However, the last but no the least, the model not only helps to minimise this cost but also helps purchase managers to reduce work time and effort. The time freed by the model allows managers to develop other activities or exploring different alternatives making them more efficient in their daily job.

### Computational performance

In this section, the computational experiments to assess the behaviour of the proposed model is presented. The algorithm to solve the model was coded in C++ using the OpenMP library (2016), the Eigen C++ library (2016) and OPL, CPLEX 12.6 C++ API (2015). Moreover, tests were conducted on a virtual scientific computing platform of the University of Lleida, known as Stormy. More information about this infrastructure can be found in Stormy (2016). The virtual machine chosen to develop the experiments was configured with 10 CPU of 2 cores each one, 25 GB of RAM and also 100 GB of HDD. The operating system used was Ubuntu 13.04.

A collection of benchmark instances, considering different possible scenarios was generated. A set of 30 farms and a set of 70 grocery shops are considered, so $F=30$ and $C=70$. The tests go from a small set of stochastic scenarios, where ${\varOmega }=20$ to a huge set of them, where ${\varOmega }=300$. Moreover, the uncertain scenarios were generated taking into account different combinations of financial and economic situations in the production and demand. Table 5 summarise the size and configuration of the whole instances. The main parameters are #Farms, number of available farms; #Grocery shops, number of customers; and ${\varOmega }$, number of stochastic scenarios.

**Table 5:** Testbench

Instance	#Farms	#Grocery shops	${\varOmega }$
S1	30	70	20
S2	30	70	50
S3	30	70	80
S4	30	70	100
S5	30	70	200
S6	30	70	300

Main configuration parameters

Table 6 shows the dimensions of the instances in the compact and splitting variable representations. This table extends the information presented in Table 5, showing the real complexity of the problems solved. The heading are as follows: *m*, number of constrains; $n_{y}$, number of 0–1 first stage variables; $n_{x}$, number of continuous second stage variables; ${\varOmega }$, number of scenarios.

**Table 6:** Model dimensions

Instance	Compact representation			Splitting variable representation			${\varOmega }$
	$m$	$n_{y}$	$n_{x}$	$m$	$n_{y}$	$n_x$	
S1	44,020	30	42,000	44,620	600	42,000	20
S2	110,050	30	105,000	111,550	1500	105,000	50
S3	176,080	30	168,000	178,480	2400	168,000	80
S4	220,100	30	210,000	223,100	3000	210,000	100
S5	440,200	30	420,000	446,200	6000	420,000	200
S6	660,300	30	630,000	669,300	9000	630,000	300

Size of instance sets

There is overwhelming evidence that **pSM** can be configured using a huge range of parameters. However, this was not the purpose of this paper. The results presented were computed using the parameters described in Table 7.

**Table 7:** Initial parameters for calibrating the **pSM** method

Instance	$\mu _0$	$\alpha _0$	Norm	G	# cores
S1	0	0.1	2	1	20
S2	0	0.9	2	2	20
S3	0	0.9	2	4	20
S4	0	0.001	2	5	20
S5	0	0.001	2	10	20
S6	0	0.001	2	15	20

*Norm* norm type, *G* cluster size

Table 8 shows the results obtained using as solver CPLEX with automatic setting and using all the available CPU’s inside the machine, solving the original S2FVPP in both the compact and splitting variable representation. The results of applying the parallel method **pSM** presented in this paper are highlighted too. The headings are as follows: $T_{CR}$, CPLEX elapsed time (seconds) for obtaining the optimal solution for S2FVPP in compact representation; $T_{SV}$, CPLEX elapsed time (seconds) for obtaining the optimal solution for 2SVPP in splitting variable representation; $T_{pSM}$ elapsed time (seconds) for obtaining the optimal solution using the **pSM** proposed in this paper; $Sp_{pSM-CR}$, the improvement in speed in the execution of the parallel method compared with the execution using the commercial solver CPLEX, solving the compact representation model; and $Sp_{pSM-SV}$, the improvement in speed in the execution of the parallel method compared with the execution using the commercial solver CPLEX, solving the splitting variable representation model.

**Table 8:** Performance of parallel Lagrangian decomposition solving the relaxed splitting variable model over the resolution of the equivalent models using CPLEX solver

Instance	$T_{CR}$	$T_{SV}$	$T_{pSM}$	$Sp_{pSM-CR}$	$Sp_{pSM-SV}$
S1	58.7	60.9	49	1.19	1.24
S2	378	386	156.74	2.41	2.46
S3	3051	3522	1419	2.15	2.48
S4	4397	4844	3568	1.23	1.43
S5	62,266	63,000	12,348	5.04	5.1

The results presented show the strengths and weakness of using a commercial solver such as CPLEX, compared with the usage of the parallel method proposed in this paper. The use of **pSM** only depends on the model size. The results provide confirmatory evidence that the method proposed is very suitable to deal with these kind of problems. These results highlight the goodness of applying parallel decomposition techniques instead of using commercial solution to deal with full stochastic models.

Small instances, such as S1 and S2 are very far from real-life problems. Whereas, the bigger the problem is, the closer to the real problems. The analysis of the biggest instances show a huge reduction in computing time, and proves the applicability and efficiency of the algorithm for dealing with the resolution of the model with real-life data. On logical grounds, there is no compelling reason to argue that instances S3, S4, or S5 are more suitable to be solved by the method proposed. The main advantage of the method proposed is the considerable improvement in computing performance, taking the computing time as a metric to compare the proposed method and CPLEX solver. Finally, the method seems to be very scalable, because the bigger the problem is, the more speed up reached.

The initial upper bound of the solution value of the original problem is obtained by using an intuitive heuristic. This heuristic obtained the greatest possible feasible solution by fixing to 1 all the values of the boolean 0–1 first stage variables and then solving the problem. To portray the issue in farms terms, the basic idea is solving the model by choosing all the farms. By this way, it is possible to obtain a feasible upper bound in a few seconds. The results presented show that this upper bound is good enough to achieve competitive results.

On the other hand, the results show that the solution of the splitting variable representation takes much more time than the compact representation for solving the model. Therefore more computation effort is needed to obtain the Lagrangian multipliers vector ($\mu _{0}$) in the case of being initialized as the dual variables of the non-anticipativity constraints. This vector is initialized to zero in order to saving time by avoiding the calibration phase of the method.

Finally, these results boost the usage of the model by purchase managers in the agroindustry because models are solved in a reasonable computing time.

## Conclusions

This work is focused on the design and resolution of a model to deal with the selection of suppliers for a chain of grocery shops. The objective of this selection is to contract the production of fresh vegetables. Hence the grocery shop can offer local products and have a better position to control quality and traceability. The model takes into account the future demand of a set of customers and the uncertain production of a farm during a season. The objective was to develop a two stage mixed 0–1 model considering uncertain production and demand. Thus, the model presented seems to be a good approach for solving this kind of problems. Moreover, the good behaviour of parallel Lagrange Decomposition for resolving the model shows its applicability to the real-life problems. The integration of the model in the software of purchase managers give them competitive advantage when contracting production.

The proposed model is very practical and flexible and it will be very easy to adapt to other contexts with the same necessities.

In the future, this model will be transformed into a fully supply chain model considering all the uncertain costs of production, transportation, storage and delivery so as to make a decision model capable of taking tactical and strategic decisions for the full vegetable supply chain.

Finally, the parallel algorithm presented in this paper can be improved using other methods to solve the Lagrangian relaxation, as a cutting plane algorithm, the progressive hedging algorithm, or the parallel combination of each of these.
